# Anomalous origin of the left coronary artery from the pulmonary artery as a rare cause of mitral valve prolapse: a case report

**DOI:** 10.1186/s12872-022-02729-z

**Published:** 2022-07-04

**Authors:** Xuefeng Wang, Xiaorong Xia, Weiyi Huang, Xin Li, Yingcai Liu

**Affiliations:** 1 Department of Cardiology, The Affiliated Hospital of Southwest Medical University, NO. 25 Taiping Street, Luzhou, 646000 Sichuan China; 2 Ultrasound Department, The Affiliated Hospital of Southwest Medical University, NO. 25 Taiping Street, Luzhou, 646000 Sichuan China; 3 Department of Cardiovascular Surgery, The Affiliated Hospital of Southwest Medical University, NO. 25 Taiping Street, Luzhou, 646000 Sichuan China

**Keywords:** Coronary artery anomaly, Mitral valve prolapse, Computed tomography angiography, Echocardiogram

## Abstract

**Background:**

Mitral valve prolapse (MVP) is an etiologically heterogeneous disorder. Early diagnosis and prompt treatment of the underlying disease are of great significance. Herein, we present a rare case of MVP caused by anomalous origin of the left coronary artery from the pulmonary artery (ALCAPA).

**Case presentation:**

A 22-year-old female presented with a 16-year history of anterior mitral leaflet prolapse. However, she had never experienced any discomfort before. At a routine follow-up, a transthoracic echocardiogram showed anterior mitral leaflet prolapse (A2) with moderate mitral regurgitation, and a retrograde blood flow from an extremely dilated left coronary artery (LCA). Further coronary angiography and coronary computed tomography angiography confirmed the diagnosis of ALCAPA. She subsequently underwent successful LCA reimplantation and concomitant mitral valve replacement. Intraoperatively, her mitral annulus was mildly dilated, anterior mitral valve leaflet appeared markedly thickened with rolled edges, and a chordae tendineae connecting the anterior leaflet (A2) was ruptured and markedly shortened.

**Conclusions:**

ALCAPA is a rare and potentially life-threatening congenital coronary artery anomaly that may cause mitral valve prolapse. Echocardiogram is an important screening tool for this disorder.

**Supplementary Information:**

The online version contains supplementary material available at 10.1186/s12872-022-02729-z.

## Background

Mitral valve prolapse (MVP) is an etiologically heterogeneous disorder [1, 2]. Early diagnosis and prompt treatment of the underlying disease are critical. Anomalous origin of the left coronary artery from the pulmonary artery (ALCAPA) is a rare and potentially life-threatening congenital coronary artery anomaly with an incidence of 1 in 300,000 live births, accounting for 0.25–0.5% of all congenital heart diseases [3]. Rarely, it is associated with other cardiac anomalies such as atrial septal defect, ventricular septal defect, aortic coarctation, tetralogy of Fallot or bicuspid aortic valve [4, 5]. Only 10% to 15% of ALCAPA patients develop significant collateral circulation from the right coronary artery (RCA) to the left coronary artery (LCA) and survive into adolescence or even adulthood with no or mild symptoms[6]. Nonetheless, collateral vessels are often insufficient to supply the entire left ventricle, resulting in ischemic dysfunction/impairment of the papillary muscles and adjacent myocardium, and ultimately mitral valve prolapse [5, 7]. Herein, we report an adult case with asymptomatic ALCAPA and anterior mitral leaflet prolapse, which was evaluated using multimodality imaging and then underwent successful reimplantation of the left coronary artery into the aorta and mitral valve replacement.

## Case presentation

A 22-year-old female was referred to our out-patient clinic for follow-up on her asymptomatic mitral valve prolapse (MVP), which was discovered incidentally when she was 6 years old. She had never previously experienced dyspnea, chest pain or syncope. Physical examination revealed a grade 3/6 systolic murmur at the upper left sternal border. Her electrocardiogram showed sinus rhythm with left anterior fascicular block and T wave inversion in leads I and aVL. All laboratory tests, including cardiac troponin, N-terminal pro-B-type natriuretic peptide (NT-pro BNP) and autoantibodies, were unremarkable.

**Fig. 1:**
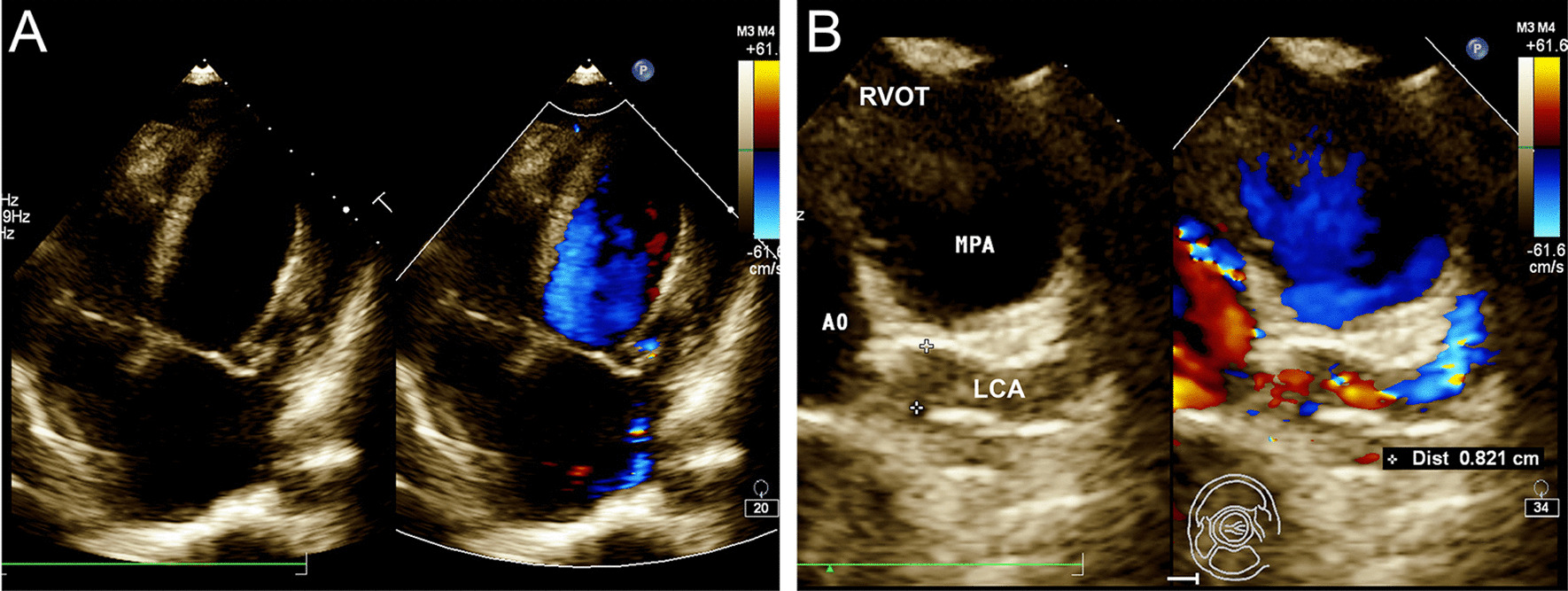
Echocardiogram showing anterior mitral leaflet prolapse (**a**) and reversed flow from an extremely dilated left coronary artery (**b**)

**Fig. 2:**
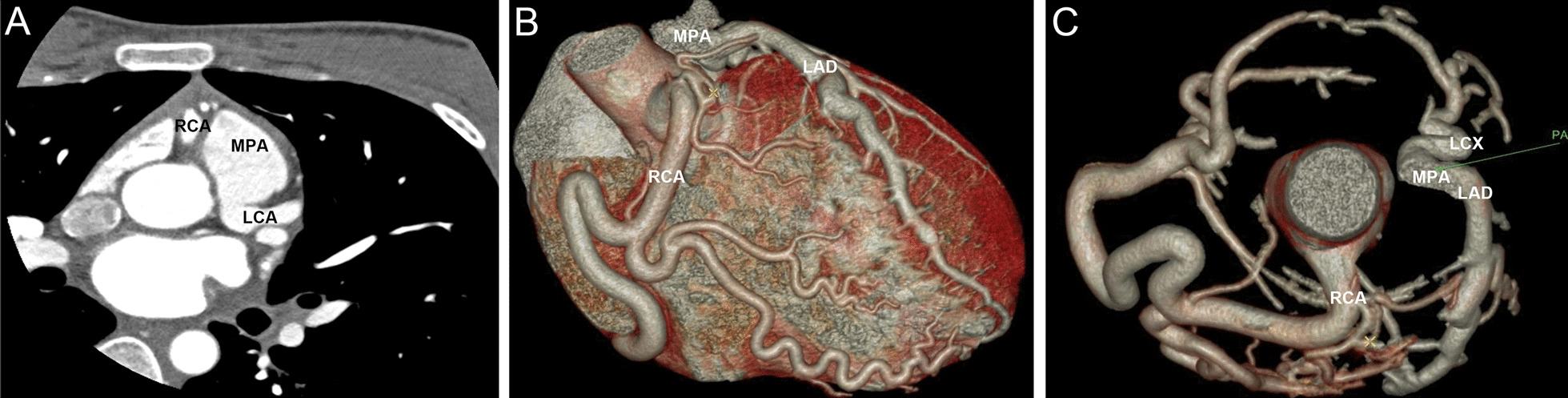
Coronary computed tomography angiography showing the origin and course of the left coronary artery

In addition to the anterior mitral leaflet prolapse (A2) with moderate mitral regurgitation and a mildly dilated left ventricle (left ventricular end-diastolic diameter: 56 mm) with a preserved ejection fraction (Fig. 1a and Additional file 1: Video 1), transthoracic echocardiogram revealed a retrograde blood flow from an extremely dilated left coronary artery (LCA) (Fig. 1b and Additional file 2: Video 2). Coronary angiography also revealed an enormously dilated and tortuous right coronary artery (RCA) originating from the right coronary cusp, with many collateral vessels filling the LCA (Additional file 3: Video 3), but failed to locate the orifice of LCA. Further coronary computed tomography angiography confirmed the diagnosis of ALCAPA (Fig. 2).

**Fig. 3:**
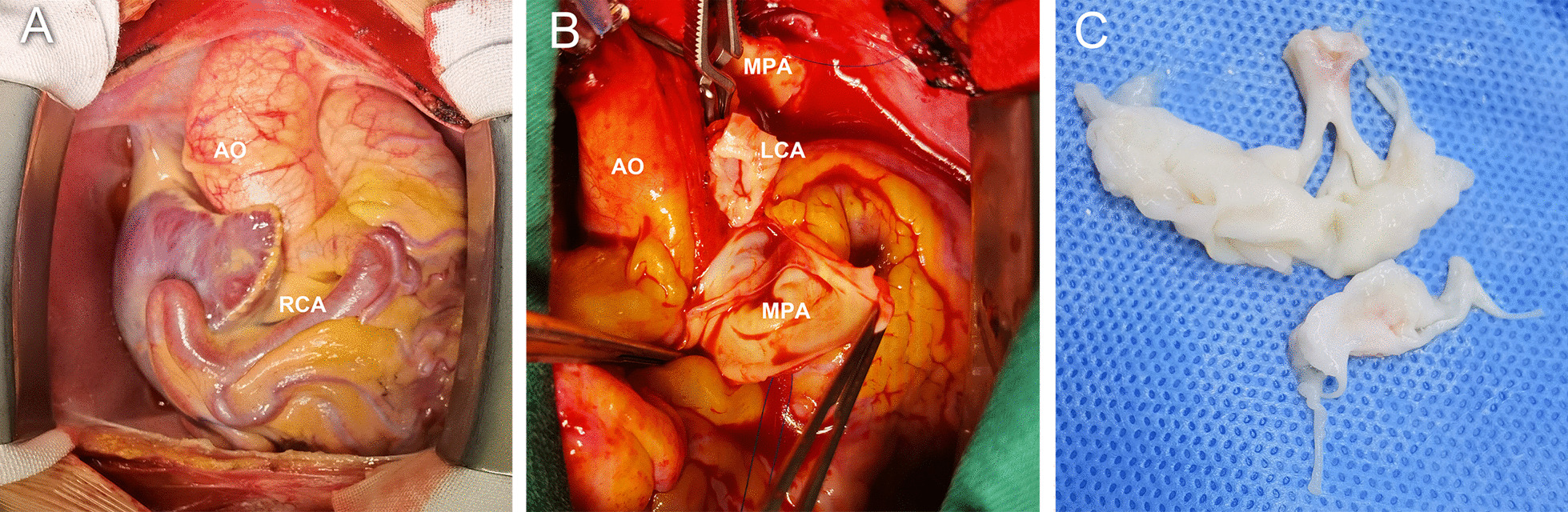
Intraoperative images demonstrating the anatomy of the right coronary artery and collateral vessels (**a**), the anomalous origin of the left coronary artery (**b**), and the impaired mitral valves (**c**)

Given her high risk of left ventricular dysfunction, heart failure and malignant ventricular dysrhythmias, surgical correction was scheduled. Intraoperatively, her coronary arteries were found to be extremely dilated, the left ventricle and mitral annulus were mildly dilated, and anterior mitral leaflet appeared apparently thickened with rolled edges. More importantly, a chordae tendineae connecting the anterior leaflet (A2) was ruptured and markedly shortened (Fig. 3A–C). Therefore, reimplantation of the LCA into the aorta and concomitant mitral valve replacement were performed. The patient recovered uneventfully and was discharged from the hospital 2 weeks later. Three months following surgery, an echocardiogram revealed that her left ventricle had returned to normal (left ventricular end-diastolic diameter: 47 mm).

## Discussion and conclusions

Anomalous origin of the left coronary artery from the pulmonary artery (ALCAPA), also known as Bland-White-Garland syndrome, is a rare and potentially life-threatening congenital coronary artery anomaly. It affects about 1 in every 300,000 live births, accounting for 0.25–0.5% of all congenital heart diseases [3]. Rarely, it is associated with other cardiac anomalies such as atrial septal defect, ventricular septal defect, aortic coarctation, tetralogy of Fallot or bicuspid aortic valve [4, 5]. Its manifestations and prognosis are strongly influenced by the magnitude of collateral vessels between the RCA and LCA. The overwhelming majority of patients with ALCAPA fail to develop significant collateral circulation from the RCA to the LCA during their early infancy period, and more than 90% of patients will suffer severe heart failure and ultimately die within the first year of life if left untreated. Only 10% to 15% of patients develop significant collateral circulation and survive into adolescence or even adulthood with no or mild symptoms [6]. Nevertheless, it is often insufficient to supply the whole left ventricle through collateral vessels, resulting in chronic myocardial ischemia. As a result, 80–90% of these patients may develop malignant ventricular dysrhythmias, the majority of which occur within the first 3 decades of life [5, 6]. In addition, they are at risk of developing silent myocardial infarction, left ventricular dysfunction and mitral insufficiency [5]. Our patient, who was also a young mother, had never experienced any discomforts in her life and her left ventricular function was preserved. This may have been attributed to her well-developed collateral vessels.

ALCAPA is most prevalent in infants, children and young adults. Therefore, this rare disorder may go unnoticed. Transthoracic echocardiogram, which is widely accessible, allows for a rapid and noninvasive assessment of structural and functional cardiac abnormalities at relatively low cost and is, therefore, the preferred imaging modality for these patients [8, 9]. In the present case, the extremely dilated coronary arteries and retrograde blood flow from the LCA shown by echocardiogram prompted us to search for the underlying cause of MVP. While coronary angiography allows for good visualization of the course of the anomalous coronary artery and collateral vessels, it is invasive and has some complications. Coronary computed tomography angiography, which has an excellent spatial and temporal resolution, can noninvasively show the origin and course of the anomalous coronary artery and it is regarded as the mainstay diagnostic technique for ALCAPA [10]. In addition, cardiac magnetic resonance imaging is a highly valuable imaging modality because of its unique tissue characterization, which enables the accurate assessment of myocardial ischemia and fibrosis [11, 12].

Prompt surgical intervention is critical for patients with ALCAPA, as it allows for gradual and even complete myocardial recovery [6, 13]. The recommended treatment for ALCAPA is direct reimplantation of LCA into the aorta to reestablish two-coronary circulation. In cases where direct reimplantation of LCA is not technically feasible (eg. LCA originating far from the aorta, abundant collaterals or noncompliant tissues around the ostium of ALCAPA), intrapulmonary tunnel repair (Takeuchi operation) or coronary artery bypass grafting (CABG) with LCA ligation should be considered [6, 14].

Mitral insufficiency (MR), a common complication of ALCAPA, is predominantly caused by ischemic dysfunction of the papillary muscles and adjacent myocardium, as well as annular dilation due to left ventricular remodeling [3, 15]. In these situations, the majority of mitral insufficiencies are reversible and may improve following ALCAPA repair, with only a small percentage deteriorating and requiring mitral valve reintervention [16]. Therefore, performing mitral valve intervention at the time of ALCAPA repair is still contentious. Nonetheless, structural abnormalities of the mitral apparatus including MVP, chordae tendineae rupture, mitral valve cleft and papillary muscle infarction/fibrosis, which occur in about 20% of ALCAPA patients, can’t recover from revascularization and concomitant mitral valve repair should be considered [3, 13, 16].

**Fig. 4:**
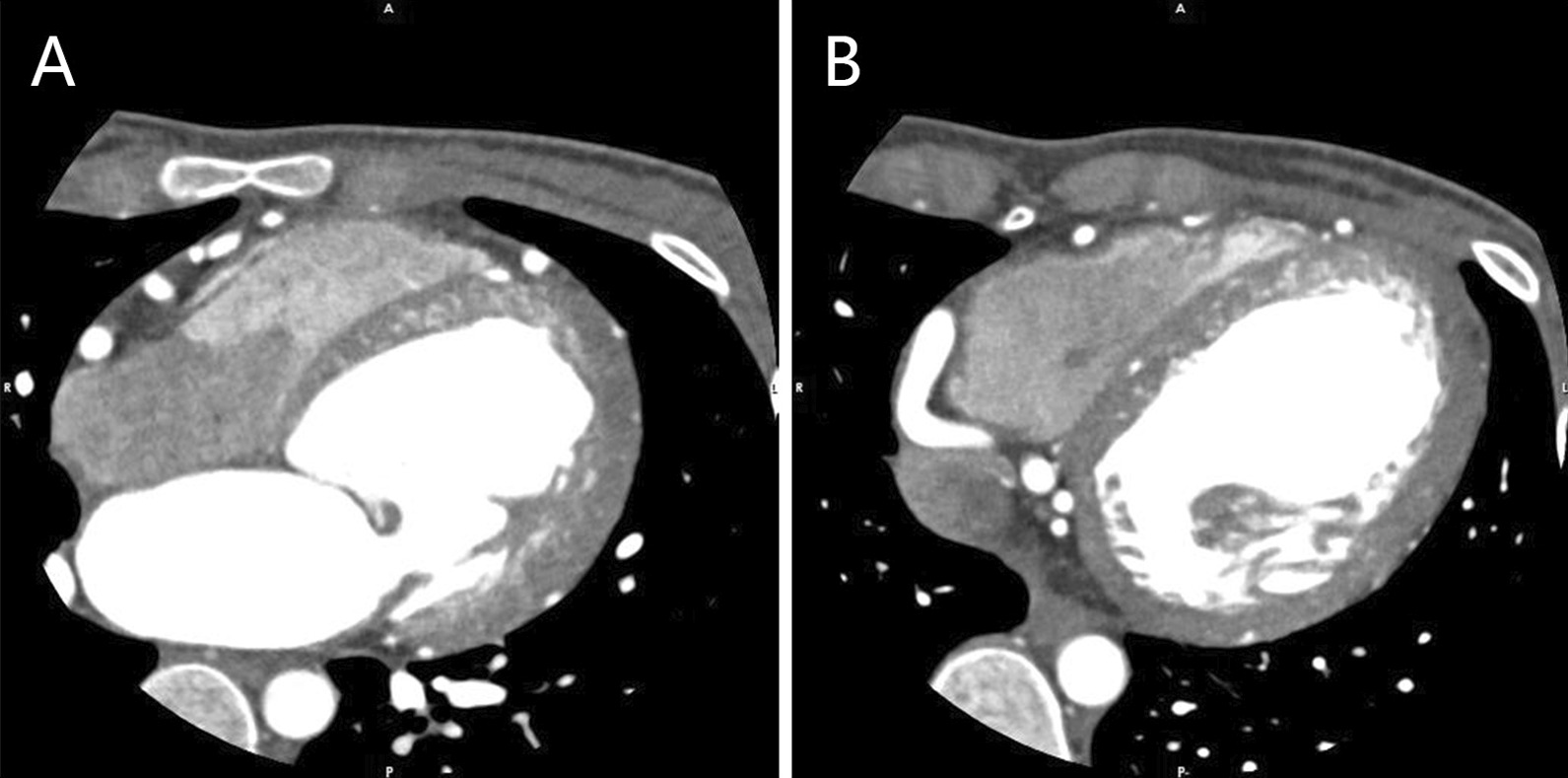
Enhanced computed tomography of the small anterolateral papillary muscle (**a**) and the normal posteromedial papillary muscle (**b**)

The mechanisms of MVP in ALCAPA may be similar to those in coronary artery disease, which include ischemic impairment of the papillary muscles and adjacent myocardium as well as chordae tendineae rupture [7, 17, 18]. Different from coronary artery disease, a recent study found that anterior leaflet prolapses occur more often in ALCAPA patients than posterior ones [16]. This may be explained by the difference in blood supply. For ALCAPA patients, the left ventricle is entirely supplied by the low-pressure collateral vessels. Additionally, the blood from collateral vessels preferentially flows into the low-pressure pulmonary circulation rather than into the high-resistance myocardial circulation, resulting in a longstanding “coronary steal” phenomenon. Lastly, the anterior papillary muscle is exclusively supplied by the remote branch of LCA [19]. In this case, the arterial supply of the anterior papillary muscle is severely compromised and the chordae tendineae connecting the anterior papillary muscle is highly vulnerable. Therefore, the ruptured chordae tendineae (A2) in our patient was probably caused by previously severe ischemic impairment. Furthermore, her anterolateral papillary muscle on the enhanced CT was much smaller than the posteromedial papillary muscle (Fig. 4), also indicating longstanding ischemic atrophy of the anterolateral papillary muscle.

In conclusion, ALCAPA is a rare and potentially life-threatening congenital coronary artery anomaly that may cause mitral valve prolapse. Echocardiogram is an important screening tool for this disorder.

## Supplementary Information

**Additional file1: Video 1 **Echocardiogram showing anterior mitral leaflet prolapse with moderate mitral regurgitation and a mildly dilated left ventricle with a preserved ejection fraction.

**Additional file 2: Video 2 **Echocardiogram showing a retrograde blood flow from an extremely dilated left coronary artery.

**Additional file 3: Video 3 **Coronary angiography showing an enormously dilated and tortuous right coronary artery with many collateral vessels filling the left coronary artery.

## Data Availability

The datasets used during the current study are available from the corresponding author on reasonable request.

## Abbreviations

ALCAPA: Anomalous origin of the left coronary artery from the pulmonary artery
LCA: Left coronary artery
RCA: Right coronary artery
LAD: Left anterior descending artery
AO: Aorta
MPA: Main pulmonary artery
RVOT: Right ventricular outflow tract
LCX: Left circumflex
